# Preemptive Direct Aortic Insertion of Impella 5.5 in Patients Undergoing Cardiac or Aortic Surgery

**DOI:** 10.1016/j.atssr.2024.06.003

**Published:** 2024-06-20

**Authors:** Masaaki Ryomoto, Masaru Ishida, Kanji Ishizu, Toshihiro Funatsu

**Affiliations:** 1 Department of Cardiovascular Surgery, Rinku General Medical Center, Osaka, Japan

## Abstract

The mortality rate of postcardiotomy cardiogenic shock after cardiovascular surgery is quite high, and the only way to avoid this serious complication is to initiate a preemptive strategy during surgery. The Impella 5.5 device with the SmartAssist system (Abiomed) is mainly used to prevent or to treat cardiogenic shock in cardiac surgery, but it is not often used in aortic surgery. We present the technique of preemptive insertion of the Impella 5.5 device through the ascending aorta intraoperatively to prevent the onset of postcardiotomy cardiogenic shock after cardiac or aortic surgery.

The mortality rate of postcardiotomy cardiogenic shock after cardiovascular surgery is as high as 50% to 80%.^1^ Once the patient is in cardiogenic shock, the clinical outcome is no longer improved even with venoarterial extracorporeal membrane oxygenation.^2^ However, a good outcome is achieved with the early initiation of Impella device (Abiomed) support. The Impella reportedly unloads the left ventricle so that the myocardial oxygen demand is significantly reduced under total support of the device.^3^

It has been reported that preemptive insertion of the Impella 5.0 or 5.5 through the axillary artery is valuable in patients with left ventricular disfunction who are undergoing heart surgery.^4^ However, it may be difficult to insert the Impella 5.5 in small-bodied patients with a small diameter of the axillary artery.

Six patients underwent preemptive Impella 5.5 insertion through the ascending aorta (direct aortic access) during their surgical procedure (3 valvular, 2 aortic, and 1 ventricular surgery). Here we report our technique to establish a standard strategy during cardiac or aortic surgery in patients with severe left ventricular dysfunction or patients requiring left ventricular systolic unloading.

## Technique

**Figure 1:**
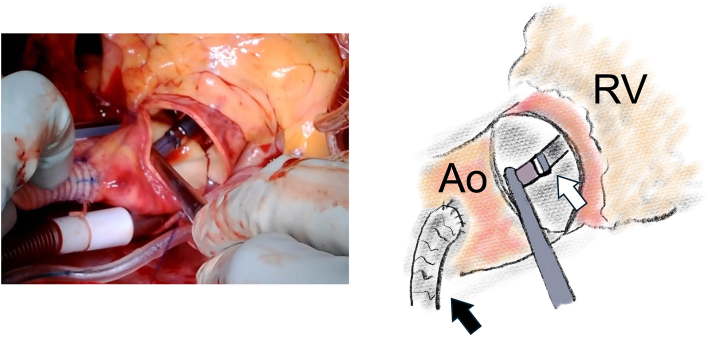
Intraoperative findings in a patient undergoing aortic valve replacement. The Impella 5.5 device (Abiomed) was introduced through the prosthesis into the left ventricle under direct vision before the aortotomy was closed. The black arrow indicates the prosthesis anastomosed to the ascending aorta (Ao) to introduce the device. The white arrow indicates the Impella 5.5 inside the ascending aorta. (RV, right ventricle.)

**Figure 2:**
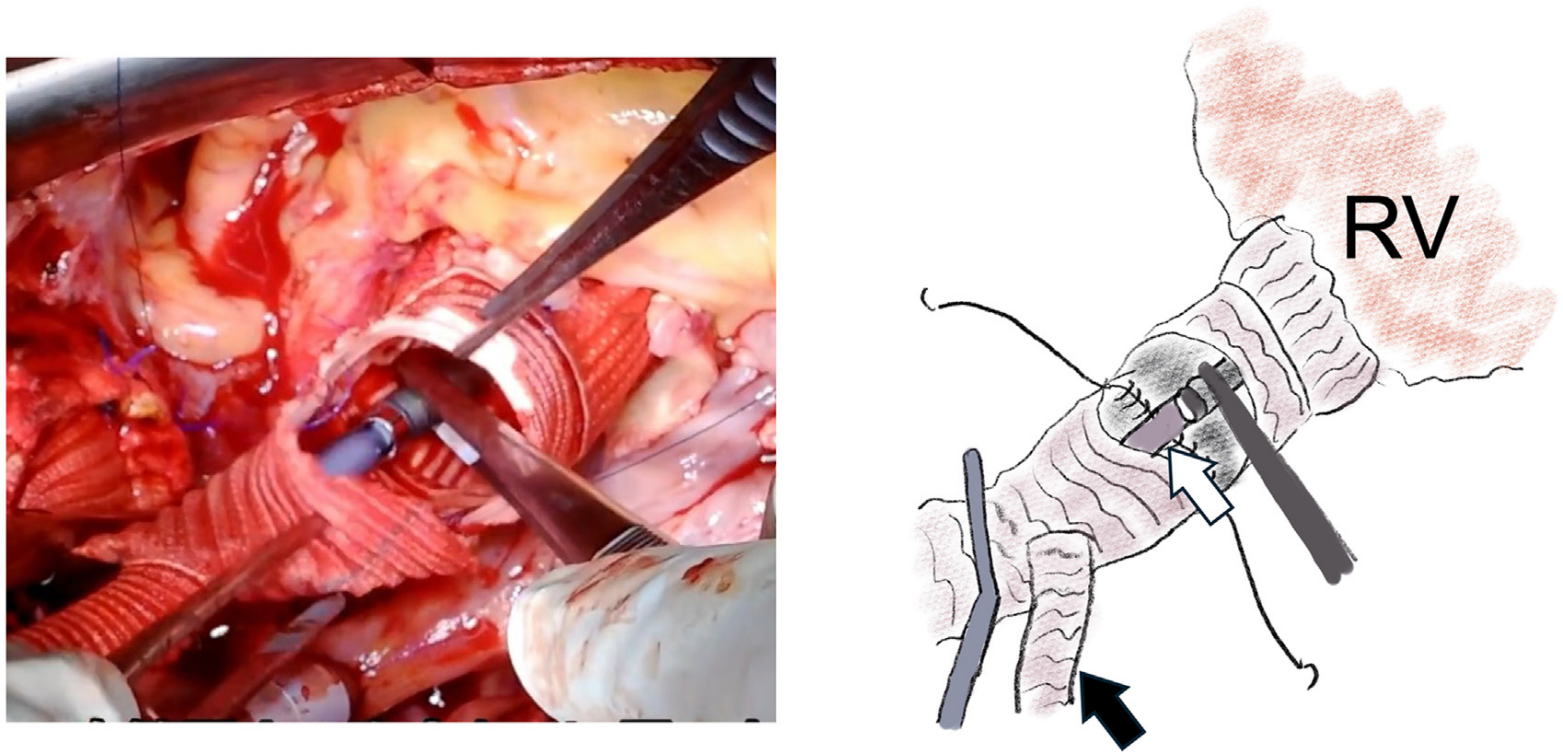
Intraoperative findings in a patient undergoing the Bio-Bentall procedure and hemiarch replacement. After the proximal and distal prostheses were anastomosed one-third circumferentially, the Impella 5.5 device (Abiomed) was introduced through the side branch of the distal prosthesis under direct vision. The black arrow indicates the side branch of the distal prosthesis, and the white arrow indicates the Impella 5.5. (RV, right ventricle.)

**Figure 3:**
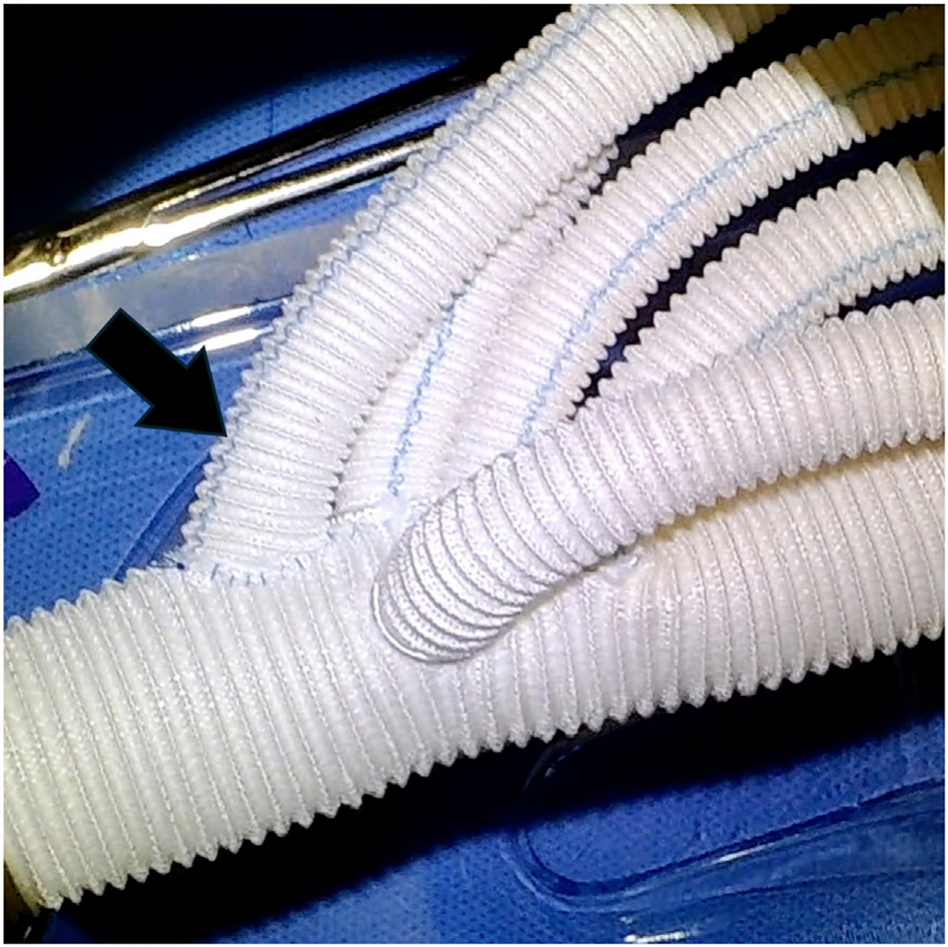
The quadrifurcated prosthesis used for total arch replacement. The side branch used to introduce the Impella 5.5 device (Abiomed) was preliminarily anastomosed to the back table near the first branch, which was used to reconstruct the brachiocephalic artery. The arrow indicates the anastomosed side branch.

The indication for preemptive Impella 5.5 insertion was preoperative left ventricular dysfunction of 30% or less or the need for left ventricular systolic unloading. The timing of insertion of the Impella 5.5 differed between surgery with or without aortotomy. In cardiac surgery with aortotomy, the 9-mm J graft (Japan Lifeline Co) was anastomosed with 5-0 polypropylene suture to the ascending aorta at a site 6 cm from the sinutubular junction during cardioplegic arrest. The Impella 5.5 was then introduced through the anastomosed graft into the left ventricle through the prosthetic aortic valve under direct vision from the aortotomy. After the station of the outlet portion was confirmed to be above the aortic valve (Figure 1), the aortotomy was closed. In aortic surgery, the Impella 5.5 was inserted through the branch of the prosthesis when the hemiarch or partial arch replacement was performed and three-fourths of the proximal anastomosis was achieved (Figure 2). The device was easily introduced into the left ventricle through the aortic valve or the bioprosthesis. If total arch replacement was performed using a quadrifurcated graft, 4-0 polypropylene suture was used to anastomose the 11-mm graft to the main graft close to the 11-mm branch in advance (Figure 3), and the device was consequently introduced through this branch (Video 1).

In surgery without aortotomy, the 9-mm graft was anastomosed with 5-0 polypropylene suture under partial aortic clamping after the cardioplegic arrest was released. The guidewire was introduced through the 9-mm graft into the left ventricle, and the Impella 5.5 was then advanced in an over-the-wire fashion under fluoroscopic guidance. The graft introducing the Impella 5.5 was passed out to the chest wall though the supraclavicular portion before the device was inserted in surgery with or without aortotomy.

The heparinization was reversed completely with protamine sulfate, and hemostasis was achieved in a routine manner. The purge fluid consisted of 500 mL of 5% glucose solution and sodium bicarbonate (25 mEq/L).^5^ No heparin was administered either systemically or into the purge line for 48 to 72 hours postoperatively. After that, heparin (10 U/mL) was added to the purge fluid.

When cardiac function was restored, the Impella 5.5 was explanted in the operating room with the patient under general anesthesia. After the device was removed, back bleeding from the graft was performed to flush any debris. The remaining graft was trimmed as short as possible, and the stump was closed with 4-0 polypropylene running suture followed by skin closure.

## Comment

The Impella 5.5 was designed to be introduced through the axillary artery or ascending aorta, but it must be inserted through a 21F cannula, a maneuver that is difficult to achieve in patients with a small axillary artery. However, the transaortic approach (direct aortic implantation) can be used in most patients, without any restrictions related to body size. This technique has already been reported in patients without aortotomy.^6^ We performed direct aortic implantation of the Impella 5.5 not only in patients without aortotomy, but also in patients with aortotomy, such as those undergoing aortic valve replacement, a Bio-Bentall operation, and total aortic arch replacement. In these situations, the Impella 5.5 can be placed in the correct position easily and precisely under direct vision through the aortotomy.

When using the direct aortic implantation technique, the hemostatic strategy is very important. Intraoperatively, protamine sulfate was administered to achieve complete reversal of the heparin effect and attain hemostasis. Furthermore, no heparin was administered either systemically or in the purge line for 48 or 72 hours after surgery. The purge fluid with sodium bicarbonate was administered according to the reported anticoagulation management.^5^

The Impella 5.5 was introduced through the right supraclavicular portion as reported,^6^ and the sternum was closed completely. Subsequently, no patient has had problems with postoperative hemostatic management.

All 6 patients were able to be weaned from the Impella 5.5 uneventfully. Our strategy of preemptive insertion of the Impella 5.5 through direct aortic implantation may be effective for patients undergoing cardiac or even aortic surgery who have poor left ventricular function or require systolic unloading of the left ventricle. However, the late postoperative results remain unclarified.
